# Page Kidney From a Subcapsular Urinoma Following Contralateral Radical Nephrectomy

**DOI:** 10.7759/cureus.15639

**Published:** 2021-06-14

**Authors:** Bright E Izekor, Chidozie Odigwe, Nimrit Goraya, Paula A Duran

**Affiliations:** 1 Internal Medicine, Baylor Scott & White Medical Center - Temple, Temple, USA; 2 Nephrology, Baylor Scott & White Medical Center - Temple, Temple, USA

**Keywords:** severe hypertension, urinoma, general nephrology, page kidney, renal cell carcinoma (rcc)

## Abstract

Page kidney is a rare cause of hypertension and kidney injury; it results from extrinsic compression of the kidney due to fluid accumulation in the subcapsular space. Hypertensive crisis may be the only presenting clinical sign in patients with Page kidney. Urinomas are a very rare cause of Page kidney with very few cases reported in the literature. Urinoma should be suspected in patients presenting in hypertensive crisis who have a history of recent abdominal trauma, genitourinary malignancy, and renal instrumentation. Patients diagnosed with Page kidney from a urinoma should be managed with the least invasive means possible.

## Introduction

Page kidney or Page phenomenon is the extrinsic compression of the kidneys by a mass. It is a rare cause of kidney injury and hypertension; as of 2009, there were 128 cases reported [1]. The most common reported cause of Page kidney is hematoma and is often associated with trauma or renal instrumentation. Other causes include lymphatic cysts, tumors, and urinomas. Urinomas are very rare causes of Page kidney. From our review, there were only four reported cases as of 2009 [1].

Here, we report a case of Page kidney from a right subcapsular urinoma in a patient with a solitary kidney. His pathology was complicated by ureteral obstruction and acute kidney injury (AKI). The patient initially required renal replacement therapy. Renal function dramatically improved following percutaneous drainage of urinoma, warranting discontinuation of renal replacement therapy.

## Case presentation

Our patient was a male in his mid-70s diagnosed with metastatic renal cell carcinoma affecting his left kidney. Imaging showed tumor invasion of his left renal vein as far as his inferior vena cava (IVC). He was sent to our facility for radical left nephrectomy. His surgery was complicated by difficult tumor resection and blood loss of over 3.5L. Bilateral renal veins were dissected along with the IVC, with his right renal vein clamped distally during the procedure. The surgery lasted two hours longer than anticipated. He was anuric postoperatively. His serum creatinine rose to 2.8mg/dL from a baseline of 1.5mg/dL. The impression was that his anuria was likely due to AKI from renal hypoperfusion secondary to blood loss and perioperative hypotension. He was started on hemodialysis, which he continued as an outpatient following discharge. Of note, a renal ultrasound done during this hospitalization showed very mild hydronephrosis of his right kidney with no evidence of perirenal fluid collection.

**Figure 1:**
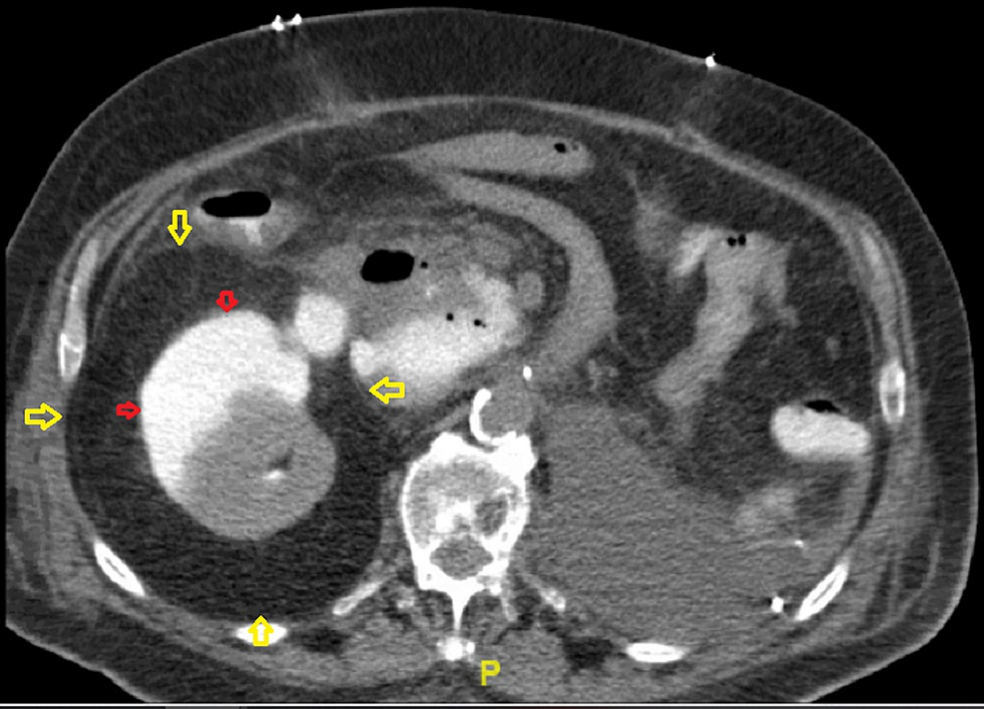
CT abdomen showing large perirenal fluid collection (yellow arrows) as well as subcapsular fluid collection (red arrows)

On postoperative day 20, he was readmitted for suspected sepsis after he presented with altered mental status. At presentation, his serum creatinine was 9.4mg/dL, while his serum potassium was 6.8mg/dL. His last dialysis was two days prior to presentation. Vitals at presentation was significant for blood pressure of 203/165; his blood pressure at discharge from his initial hospitalization was 143/85. Abdominal CT showed a large retroperitoneal hematoma and a right perinephric fluid collection measuring 11cm x 5.6cm and 17.6cm craniocaudally (Figure 1).

**Figure 2:**
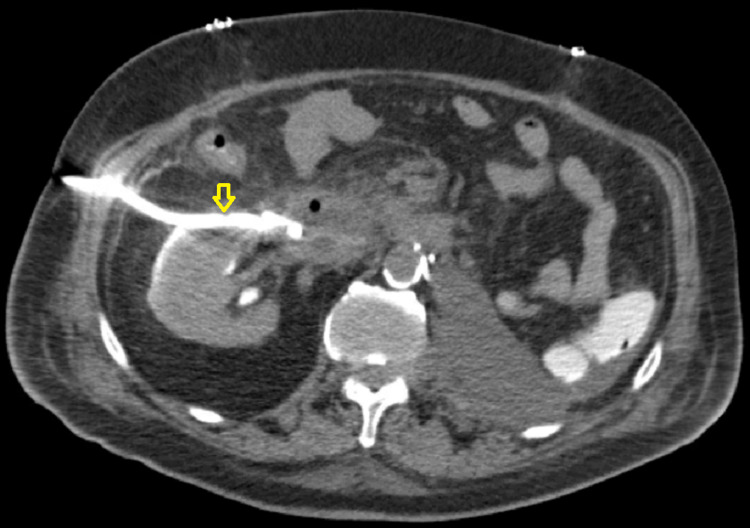
Repeat CT abdomen showing percutaneous drainage tube placement (yellow arrow)

The initial suspicion was that this perinephric fluid was likely an abscess. However, interventional radiology (IR) drainage revealed 1200mL of straw-colored fluid with analysis revealing creatinine content that was above assay limit (>74mg/dL), consistent with a urinoma. A percutaneous drainage tube was placed (Figure 2).

**Figure 3:**
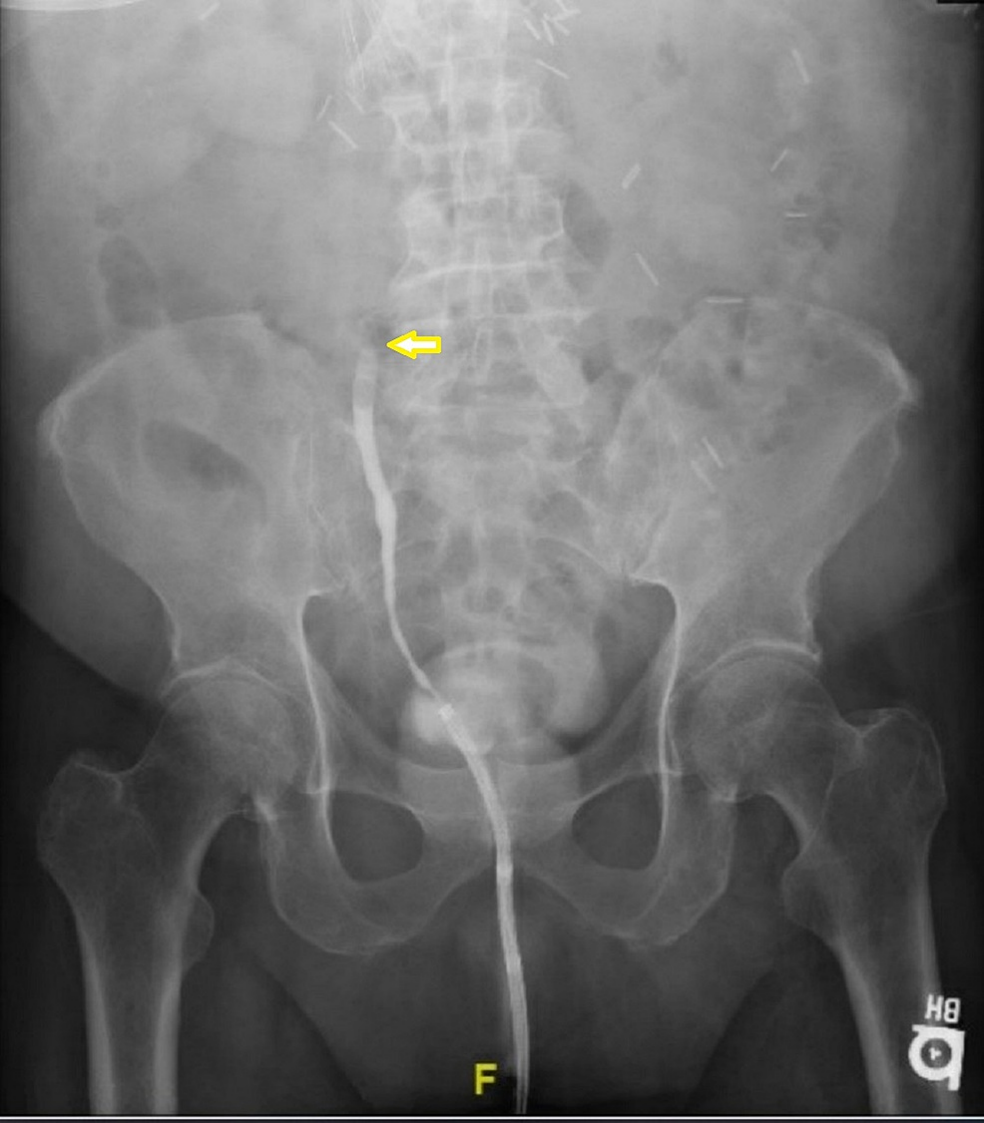
X-ray cystogram showing lack of contrast extension beyond patient's mid ureter (indicated by arrow)

Nephrostogram done from the drainage tube demonstrated a non-dilated renal collecting system, suggesting communication between urinoma and the right renal collecting system. Daily urine output from the tube was at least 1L. The patient’s renal function improved dramatically; serum creatinine was at baseline within four days of urinoma drainage and tube placement. Hemodialysis was discontinued on day one post-procedure. An x-ray cystogram showed no contrast extending beyond his mid ureter, consistent with ureteral obstruction (Figure 3).

The drainage tube was then removed, and a nephrostomy tube was placed. The plan was to further investigate his ureteral obstruction with the hope of eventual recanalization, but he opted for hospice care and was placed on comfort measures. The patient was discharged on home hospice care; he died six days post-discharge. An autopsy was not performed, so the exact cause of death is unknown. Serum creatinine at discharge was 0.8mg/dL.

## Discussion

Page phenomenon was first described in 1939 when Page wrapped canine kidneys in cellophane; the response measured was hypertension [2]. This observed hypertension was believed to be secondary to activation of the renin-angiotensin-aldosterone system (RAAS). Activation of the RAAS was theorized to be secondary to renal ischemia from renal parenchymal compression [3]. The first human case of Page kidney was reported in 1955 in a 19-year-old athlete who suffered trauma to his flank [4]. Since then, several cases of the Page phenomenon or Page kidney have been described in the literature, most commonly from a subcapsular hematoma. Other reported causes are large simple cysts, retroperitoneal paragangliomas, urinomas, and lymphoceles [1].

Page kidney from a subcapsular urinoma is rare. From our literature review, there have only been four reported cases as of 2009 [5]. The first case was documented by Patel et al. in 1984 [6]; it involved a child born with a posterior urethral valve. The other cases involved patients with blunt force trauma and constrictive perinephritis [6-8]. In this case, it was theorized that the etiology of this patient’s Page kidney was likely a ruptured fornix/calyx from ureteral obstruction and/or surgery in the setting of a solitary kidney. Possibly, the patient’s ureteral obstruction was not new but only became apparent after his left nephrectomy. Only mild hydronephrosis was seen on imaging, likely due to an alternate drainage path created by ruptured renal fornix/calyx.

As mentioned earlier, x-ray cystogram findings were consistent with mid-ureteral obstruction. Further investigation to determine the cause of obstruction was intended but not done as the patient opted for hospice care. It was theorized, however, that this obstruction was likely due to invasion by malignancy. Other possible causes, although considered unlikely, are ureteral stricture, ureteral stones, and complications from his radical nephrectomy.

Page kidney, by definition, involves hypertension due to the mechanism described above. Our patient presented with a significant elevation of his blood pressure, further supporting our diagnosis. He also presented with acute kidney injury as defined by the Kidney Disease: Improving Global Outcomes (KDIGO) guidelines [9]; therefore, he met the criteria for hypertensive emergency. There have been few cases reported of AKI resulting from Page kidney. In this case, however, his postoperative AKI was likely prerenal either from hemorrhage or perioperative hypotension. His subsequent kidney injury was deemed likely postrenal from ureteral obstruction. Though urine studies were not done to provide biochemical evidence of this conclusion, the rapid improvement of his renal function following drainage tube placement does support our assumption. It is believed that the tube created an alternate drainage path, thereby relieving the obstruction.

Subcapsular urinoma should be suspected in patients with hypertensive crises who are at high risk for Page kidney. High-risk patients include those with a history of trauma, renal instrumentation (biopsy, surgery, extracorporeal shockwave lithotripsy), renal cysts, and malignancy. Such patients should be evaluated with imaging and treated as indicated. Evaluation usually involves ultrasonography and/or CT scanning, with ultrasonography reportedly being less sensitive than CT [4]. Treatment for Page kidney initially involved performing a radical nephrectomy or open surgical evacuation. More recent treatment strategies include percutaneous drainage, endoscopic intervention, and laparoscopic renal decortication of compressing lesions, as well as medical management of hypertension [1,4,10].

## Conclusions

Page kidney should be suspected in patients with known genitourinary pathology presenting in hypertensive crisis, with or without AKI. Evaluation should be with a high index of suspicion for urinoma in patients with a history of recent abdominal trauma, renal instrumentation, and known genitourinary malignancy. Patients diagnosed with Page kidney should be managed with the least invasive means possible.
